# The ESICM’s digital revolution: Academy Critical Care Education (ACE) courses

**DOI:** 10.1186/s44158-023-00128-0

**Published:** 2024-01-10

**Authors:** Mo Al-Haddad, Joana Berger-Estilita, Nathan D. Nielsen

**Affiliations:** 1 Intensive Care Unit, Queen Elizabeth University Hospital, Glasgow, Lanarkshire UK; 2 University of Glasgow, Glasgow, UK; 3 Institute of Anesthesiology and Intensive Care, Hirslanden Medical Group, Salemspital Bern, Switzerland; 4 Institute for Medical Education, University of Bern, Bern, Switzerland; 5 CINTESIS@RISE, Faculty of Medicine, Centre for Health Technology and Services Research, University of Porto, Porto, Portugal; 6 Division of Pulmonary, Critical Care and Sleep Medicine & Section of Transfusion Medicine and Therapeutic Pathology, University of New Mexico, Albuquerque, NM USA

**Keywords:** eLearning, Virtual learning, Online learning, ESICM Academy, ESICM ACE course

## To the Editor,

The digital revolution has seen healthcare professionals reach—often exclusively—for electronic resources for information, evidence, and guidelines. In this letter, we describe the European Society of Intensive Care Medicine (ESICM)’s (https://www.esicm.org) own digital revolution: Academy Critical care Education (ACE) courses hosted on the ESICM Academy (“The Academy”) (https://academy.esicm.org/). Our aim is to share the development process of the ESICM ACE courses to raise awareness of the trove of knowledge and learning opportunities that ACE courses provide, to acknowledge the contributions of the hundreds of authors and collaborators that contributed to the ACE courses, and to hopefully inspire others to replicate the project.

ACE courses offer easily accessible and low carbon footprint education for tens of thousands of healthcare professionals caring for critically ill patients worldwide including those in low- and middle-income countries. The nucleus of the ACE courses was the Patient-Centred Acute Care Training (PACT) modules, published in 2003–2005 [1]. Noting the rapidly changing landscape of evidence and digital capabilities, the ESICM embarked on a mission to modernise its eLearning provision. In 2015, the ESICM eLearning Committee’s ambition was to create a digital resource that would be up-to-date and comprehensively cover the knowledge domain of the Competency-Based Training in Intensive Care Medicine (CoBaTrICE) curriculum (www.cobatrice.org/). The work to develop ACE courses began in earnest in 2017.

Each ACE course (free for ESICM members) is an educational package of intended learning outcomes, a CoBaTrICE mapping list, text, images, tables, and multimedia content with clickable in-line references and assessments in the style of the European Diploma in Intensive Care Medicine. The ACE course catalogue recently completed its first comprehensive review and update, and further annual updates are incorporated into the Academy’s Standard Operating Procedures.

**Fig. 1:**
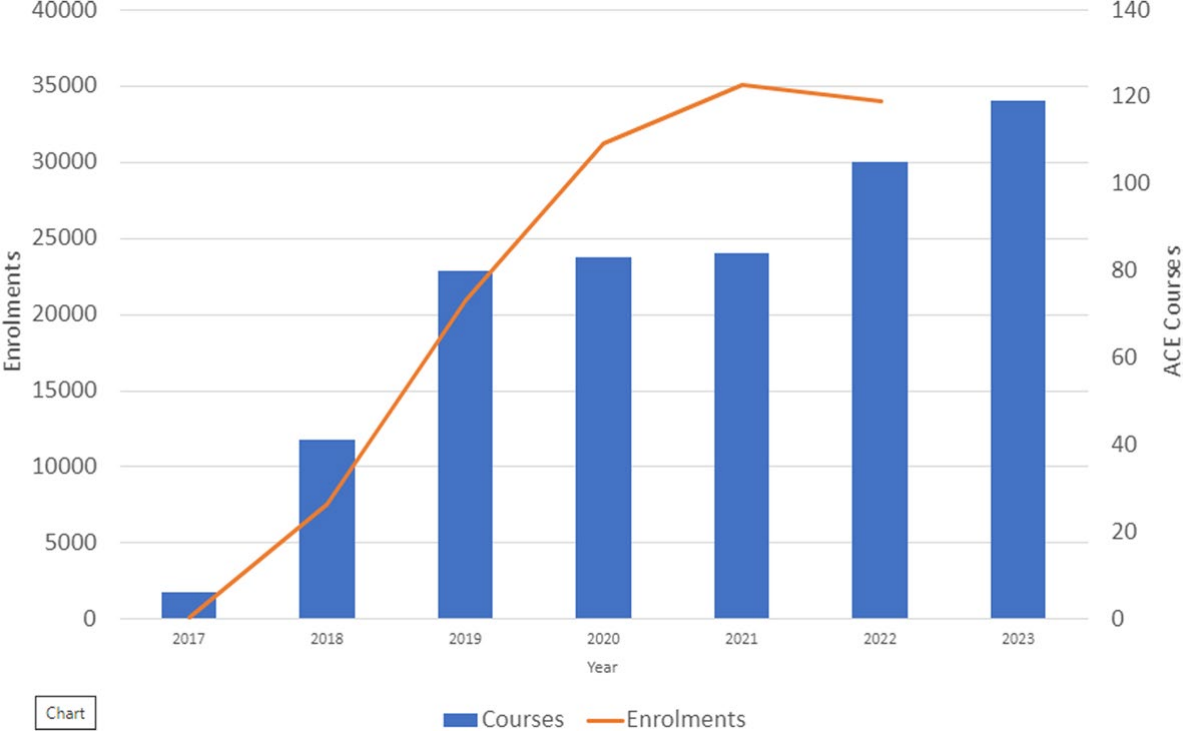
The growth of the ESICM Academy over time charted by number of ACE courses published and number of enrolments in ACE courses. The number of enrolments in 2022 is up to the 14 November

Enthusiastic educators, international authors and collaborators, rigorous peer review, standardised and lean communication, and a SCRUM™ (SCRUM.org) style of project management that helped contributors deliver value incrementally in a collaborative manner all resulted in an explosion in the number of courses available on the Academy platform (Fig. 1). The authors were mostly sought from the membership of the ESICM specialty sections and were chosen either because of their expertise in education or in the subject matter. The SCRUM™ style of project management meant that small groups worked independently and in parallel, leading to the delivery of the final product in the most efficient and timely manner. The small groups gave feedback to the ESICM Academy leadership and ultimately the eLearning Committee and chair.

The Academy received accreditation by the European Union of Medical Specialists (UEMS) in 2018. As of the time of writing, there are 257 contributing authors to the ACE courses, spanning 6 continents. Following the recent restructuring of ESICM committees, a representative from each scientific section on the eLearning Committee is now responsible for ACE courses’ content within their purview.

The link between ACE courses and CoBaTrICE is now digitised. There are established two-way collaborations with a wide range of ESICM Digital Courses and Learning Pathways. For example, four pre-course modules from the ESICM’s Research Learning Pathway have been adapted into the first research-oriented ACE courses in the Academy. The ESICM’s Academy is host to a wide range of other learning pathways and education courses including those for exam preparation.

The Academy started with a modest infrastructure—a single service on a single server—and gradually expanded its scope, embracing growth and technological advancements, until it evolved into a comprehensive and dynamic platform. It is poised to undergo significant advancements driven by the evolution and convergence of education and technology. We anticipate the integration of advanced simulation techniques and virtual reality experiences, where learners will be able to engage in realistic, immersive simulations of critical care scenarios, enhancing their clinical reasoning skills and decision-making abilities [2].

Plans are afoot to develop features that will enhance collaborative learning and social engagement such as discussion forums, live chats, and virtual study groups to facilitate international peer-to-peer learning and create a sense of community within the platform. The platform will continue to leverage data-driven learning analytics to gather insights on learners’ needs, performance, and engagement.

We anticipate that emerging technologies such as augmented reality, AI-powered chatbots, and Learning Record Stores [3] will be seamlessly integrated into the platform. We also anticipate extending the platform’s global reach by offering content and resources in multiple languages and facilitating knowledge exchange among healthcare professionals worldwide.

In conclusion, we have described the processes and outcomes of developing ESICM ACE courses. This model of education provision is sustainable, flexible, cost-effective, far reaching—including to areas with low resources—and can easily be adopted by other health professions societies.

## Data Availability

Not applicable.

## References

[1] Fluit C, Phelan D, Brown K, Ramsay G (2003). PACT: an ESICM multidisciplinary distance learning programme for intensive care training. J Intensive Care Soc.

[2] Makris D, Tsolaki V, Robertson R, Dimopoulos G, Rello J (2023). The future of training in intensive care medicine: a European perspective. J Intensive Med.

[3] Komorowski M, del Pilar Arias López M, Chang AC. How could ChatGPT impact my practice as an intensivist? An overview of potential applications, risks and limitations. Intensive Care Med. 2023;49:844–7. 10.1007/s00134-023-07096-7. 10.1007/s00134-023-07096-7 37256340

